# Operational Modal Analysis, Testing and Modelling of Prefabricated Steel Modules with Different LSF Composite Walls

**DOI:** 10.3390/ma13245816

**Published:** 2020-12-20

**Authors:** Maria Rashidi, Pejman Sharafi, Mohammad Alembagheri, Ali Bigdeli, Bijan Samali

**Affiliations:** 1 Centre for Infrastructure Engineering, Western Sydney University, Sydney 2747, Australia; M.Rashidi@westernsydney.edu.au (M.R.); P.Sharafi@westernsydney.edu.au (P.S.); B.Samali@westernsydney.edu.au (B.S.); 2 Department of Civil and Environmental Engineering, Tarbiat Modares University, Tehran 14115111, Iran; alibigdeli@modares.ac.ir

**Keywords:** modular steel frame, composite wall systems, LSF composite walls, experimental study, operational modal analysis, system identification

## Abstract

The modal properties of modular structures, such as their natural frequencies, damping ratios and mode shapes, are different than those of conventional structures, mainly due to different structural systems being used for assembling prefabricated modular units onsite. To study the dynamic characteristics of modular systems and define a dynamic model, both the modal properties of the individual units and their connections need to be considered. This study is focused on the former aspect. A full-scale prefabricated volumetric steel module was experimentally tested using operational modal analysis technique under pure ambient vibrations and randomly generated artificial hammer impacts. It was tested in different situations: [a] bare (frame only) condition, and [b] infilled condition with different configurations of gypsum and cement-boards light-steel framed composite walls. The coupled module-wall system was instrumented with sensitive accelerometers, and its pure and free vibration responses were synchronously recorded through a data acquisition system. The main dynamic characteristics of the module were extracted using output-only algorithms, and the effects of the presence of infill wall panels and their material are discussed. Then, the module’s numerical micromodel for bare and infilled states is generated and calibrated against experimental results. Finally, an equivalent linear strut macro-model is proposed based on the calibrated data. The contribution of this study is assessing the effects of different infill wall materials on the dynamic characteristics of modular steel units, and proposing simple models for macro-analysis of infilled module assemblies.

## 1. Introduction

Modular steel frames (MSFs) are a popular form of modular construction with efficient, sustainable and affordable methods [1,2]. The steel modules fabricated in the form of volumetric pre-finished units with infill wall panels are popular in mid-to-high-density developments. Despite employing the same material, their structural performance and dynamic properties, such as natural frequencies, damping ratios and modal shapes, are not the same as their conventional counterparts, mainly due to different constructional details and assembly methods. Although there is a broad range of research studies and technical reports about the modal properties of different construction methods and technologies, the structural response of MSFs is an ongoing research field [3,4,5].

Composite light steel framed (LSF) walls, including LSF frames, to which infill panels are attached, are a popular infill wall system in construction with MSFs [6,7]. This common type of walls results in a light, efficient and relatively cost-effective wall system, which is broadly used in practice. Depending on the position of each module in a MSF, different materials may be used as infill panels. For example, the interior walls are usually made from lightweight gypsum plasterboards, while for the exterior walls, which are exposed to severer environmental conditions, cement-boards or concrete panels may be utilized [8]. In general, the composite LSF wall systems are attached to the modular steel frame using screwed tracks and studs. Although this results in a coupled frame-wall system, the infill walls are usually considered non-load bearing components in the analysis and design of MSFs. The main modular steel frame is designed to sustain all the design loads. The infill walls, however, can significantly alter the dynamic properties of steel modules, which in turn may totally change the dynamic responses and consequently the entire design; especially in mid-rise and high-rise buildings [9].

The main dynamic characteristics of modular steel units, such as their natural frequencies and modal damping ratios, can be extracted through the Operational Modal Analysis (OMA) technique [10,11]. In this technique, the output-only algorithms, such as the Enhanced Frequency Domain Decomposition (EFDD) method, may be applied on pure ambient vibrations, or randomly generated vibrations of a considered steel module [12]. The ambient dynamic vibrations of a volumetric steel module can be recorded using proper sensors, mainly accelerometers, which can then be used for system identification procedures. These dynamic characteristics are being effectively employed for numerical model verification and calibration.

The effects of infill walls on the dynamic response characteristics of building frames have been previously studied where a steel frame model with mortar wall panels in plywood forms was experimentally tested [13]. The results indicate that addition of imperfectly-fitting infill walls increases the damping, and greatly influence the fundamental frequency. A shaking table test of a two-story full-scale steel frame with autoclaved lightweight concrete external wall panels showed that exterior wall panels’ contribution to the steel frame stiffness can reach up to 25% and the damping ratio can exceed by 7% [14]. Another study on the contribution of different partition walls in dynamic response buildings indicated that stiff masonry partitions had a significant effect on the response of the frame, while stud prefabricated timber partitions were more flexible and had little influence on the frame [15]. An experimental program was carried out to evaluate the dynamic responses of cold-formed steel framed gypsum partition walls. In-plane quasi-static and dynamic tests were conducted on 36 partition walls constructed using common construction details. Variables examined included framing thicknesses, stud connections to top and bottom tracks, wall intersection details, and partial height walls [16]. Gad et al. [17] studied the performance of structures with cold formed steel frames, when subjected to earthquake loading. The research involved an extensive dynamic testing program to assess the contributions from the non-structural components, particularly the plasterboard lining. The results of this study and some other similar studies show that non-structural components, such as plasterboard lining, make a significant contribution to the lateral bracing of the frames [17,18,19,20,21].

Restrepo et al. [22] presented the results of quasi-static racking tests on gypsum wallboard sheathed light gage metal stud partition walls. They studied several variables such as the configuration of the specimen, the stud thickness and spacing, the presence of a vertically slotted track at the top of the partition wall, and the wallboard thickness. Macillo et al. [23] conducted shake table tests on a full-scale two-storey building, where the mock-up was tested in two different conditions. In the first condition, the mock-up included mainly structural components of walls, floors and roof, whereas in the second condition it was completed with all non-structural components. They presented the results with respect to dynamic identification (fundamental period and damping ratio). Pnevmatikos and coworkers generated fragility curves for steel modular units and applied of control systems to reduce the vibration of these structures [24,25].

There is no specific research to date regarding the experimental dynamic testing of modular buildings to the authors’ knowledge. As this type of construction is becoming widespread across the world, the necessity of finding their natural dynamic properties is an essential part in the design stage. Especially, as steel module may be produced with infill walls as a single unit, the effects of infill panels must be considered as well. In this study, a volumetric modular steel unit is experimentally tested using Operational Modal Analysis (OMA) technique under pure ambient vibrations, and randomly generated artificial hammer impacts. The module is tested first in bare (frame only) conditions, then its edges are filled with LSF composite walls with different configurations of gypsum and cement-boards. The coupled module-wall system is instrumented with sensitive accelerometers, and its vibration responses are synchronously recorded through a data acquisition system. The main dynamic characteristics of the module are extracted using output-only algorithms, and the effects of the presence of infill wall panels and their material are discussed. Then the numerical micromodel of the module for both bare and infilled states are generated and calibrated against experimental results. Finally, an equivalent linear strut macro-model is proposed based on the calibrated data.

## 2. Testing Setup and Design of Specimens

**Figure 1:**
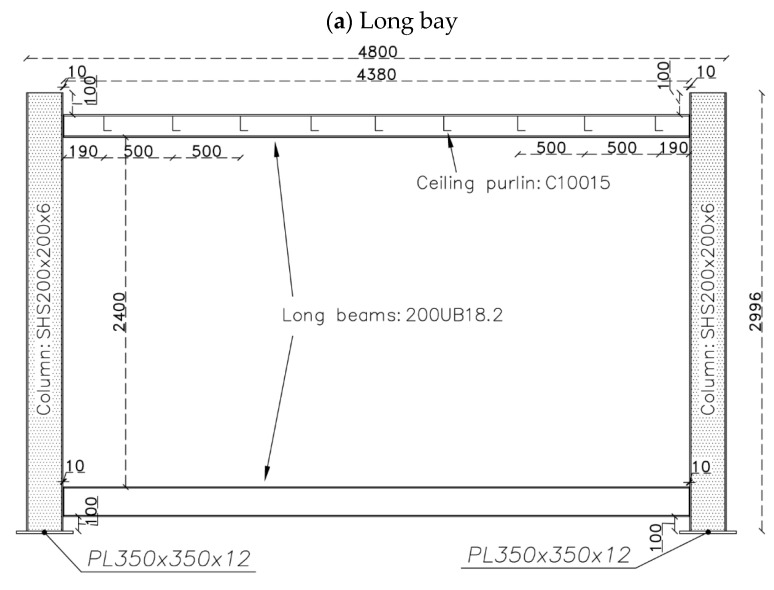

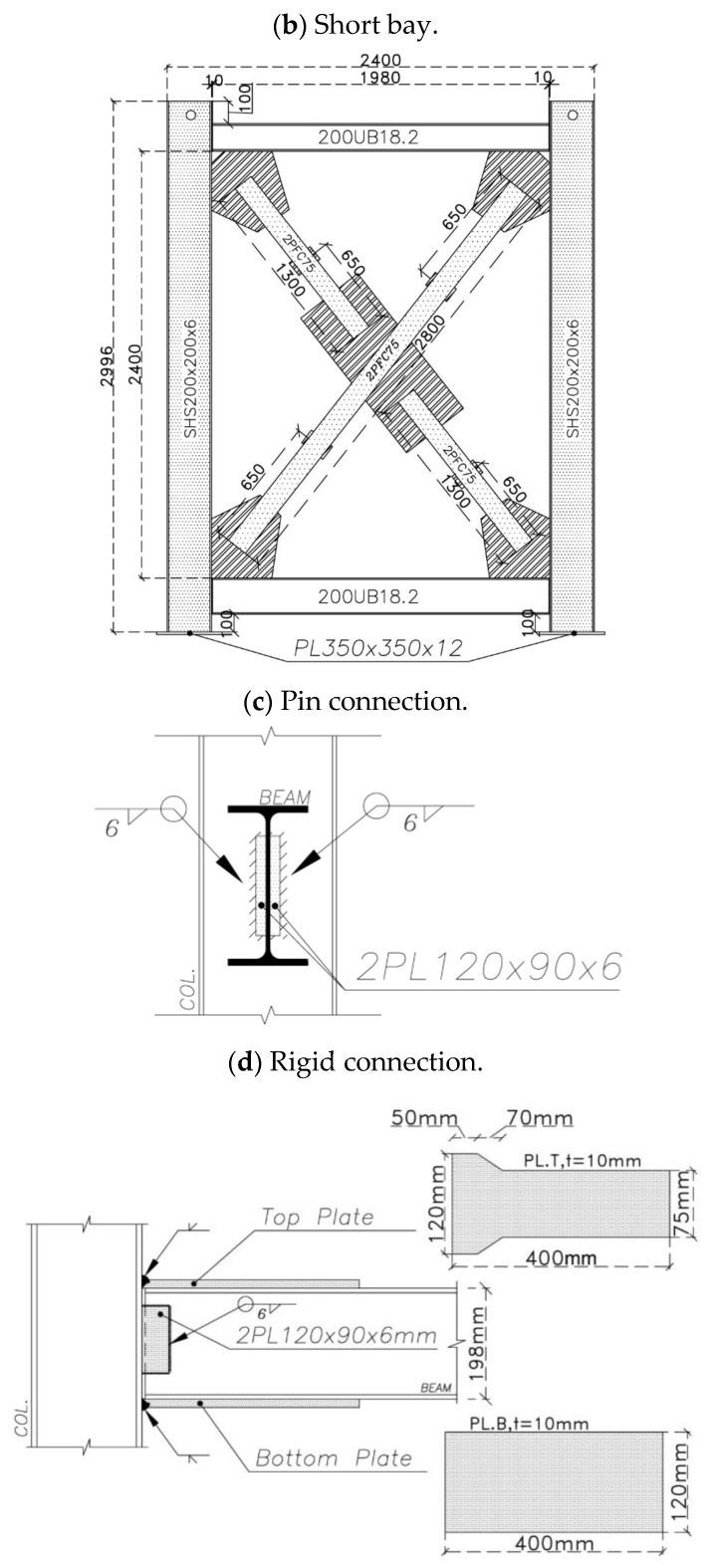

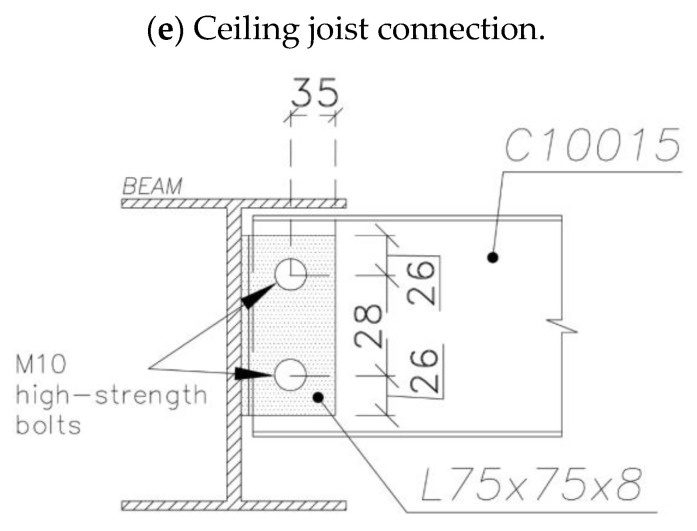
Design drawings of the steel module, all dimensions are in mm. (**a**) Long bay; (**b**) Short bay; (**c**) Pin connection; (**d**) Rigid connection; (**e**) Ceiling joist connection. All dimensions are in mm.

**Figure 2:**
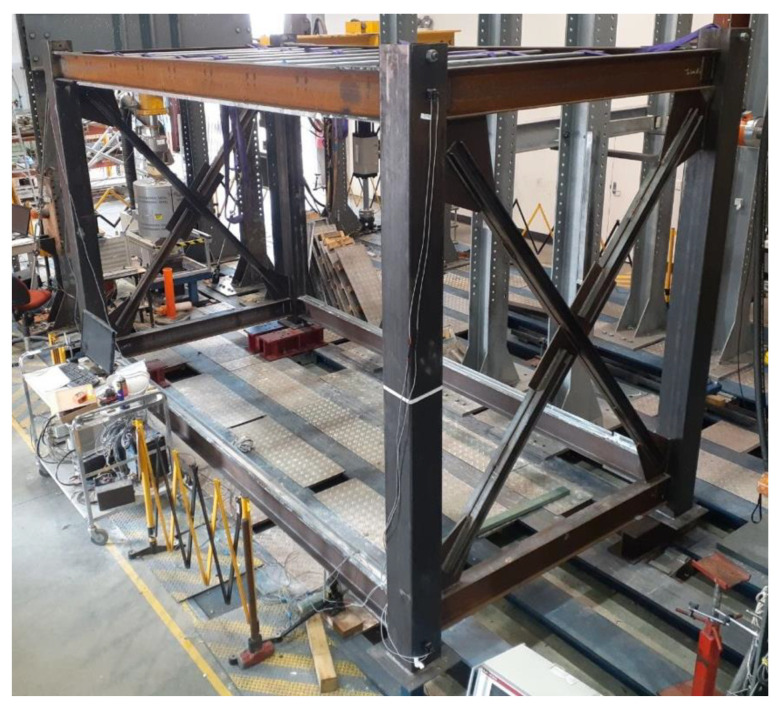
Fabricated module fixed to the strong floor.

The main testing specimen is a full-scale modular steel unit, with a total length of 4.8 m, width of 2.4 m and height of 3.0 m. The steel module was designed according to the Australian Standards AS1170 [26] and AS4100 [27], assuming to be located at the base storey of a typical six-storey corner-supported MSF. The design details are illustrated in Figure 1. The module was fabricated and fixed to the strong floor at the Structural Testing Lab of Centre for Infrastructure Engineering (CIE) at Western Sydney University. The columns were SHS200 × 200 × 9 and the beams were 200UB18.2 at both the ceiling and floor levels. The module had cross-bracing in the short span with double back-to-back PFC75 sections. The gusset plates were 8 mm in thickness. All connections were simple web plate connections, except for the long bay’s beam-column connections at the floor level, which are rigid connections with additional bottom and top plates. The ceiling joists are light steel C10015 sections at a regular space of 500 mm. They were connected to the floor beams’ web with a 75 × 75 × 8 angle along with two M10 high strength bolts. The fabricated module placed inside the CIE lab is shown in Figure 2.

**Figure 3:**
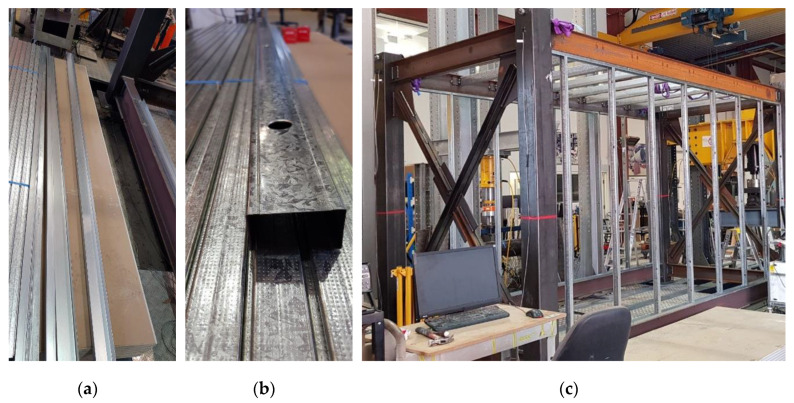
(**a**) Steel tracks, (**b**) studs, and (**c**) completed LSF assembly.

The infill wall used was the LSF composite wall system in which the infill linings with different materials were attached to the cold-formed LSF. The LSF consisted of 64 mm U-shaped galvanized tracks and studs as shown in Figure 3a,b. First, the tracks were screwed to the flanges of the top and bottom longitudinal ceiling and floor beams. Heat glue was used along the edges of the tracks for better connection to the steel flanges. Then, the studs were placed upright between the tracks at a regular distance of 600 mm, and screwed to them using self-screws. Only the module’s long bays were covered with composite wall system. The completed LSF assembly inside the long bays is shown in Figure 3c.

**Figure 4:**
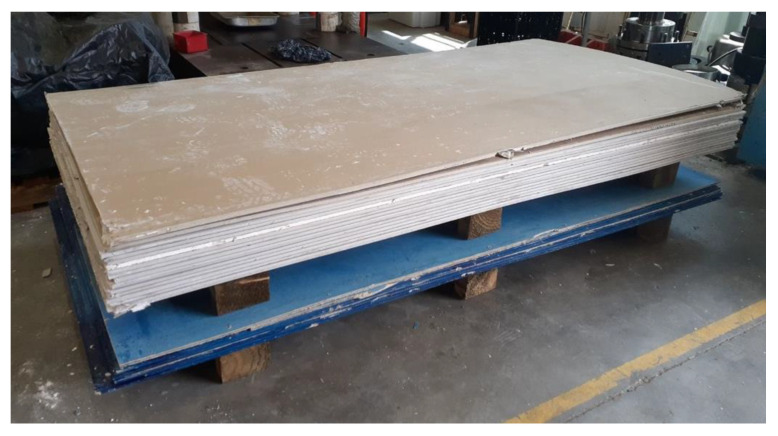
Infill panels: (top) gypsum plasterboards, (bottom) cement- or blue-boards.

**Figure 5:**
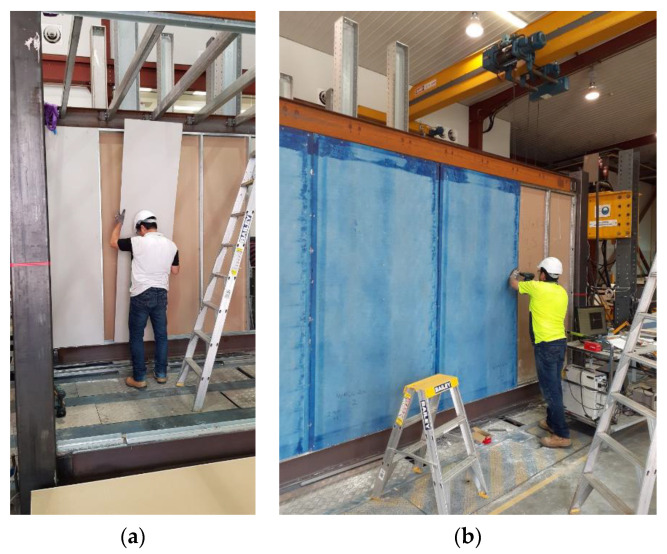
(**a**) Representative interior wall with gypsum plasterboards on both sides of the studs, (**b**) exterior wall with gypsum-board on inner side and cement-board at outer side.

After the LSF was mounted, the infill panels were firmly connected to the tracks and studs were screwed at 300 mm on centre spacing to form a complete coupling between the steel module and the infill walls. As illustrated in Figure 4, the infill panels used in this study were 10-mm-thick gypsum plasterboard linings, as well as 7.5-mm-thick cement-board (blue-board) sheets. The infill panels were mounted on both sides of the studs in each bay to form a double-sided sheathed wall comprising different interior and exterior layouts. The gypsum plasterboards attached on both sides of studs resemble an interior partition wall as shown in Figure 5a, while the cement-boards (or blue-boards) installed at outer side of the studs, represented an exterior wall, as shown in Figure 5b.

To study the effects of infill panel material, different layout configurations of interior and exterior walls were examined, listed in Table 1. In the II case, both long bays of the module were covered with the interior wall. In the case IE, one long bay was covered with the interior and another bay is covered with the exterior wall. Finally, in the EE case, both bays were filled with the exterior walls.

## 3. Instrumentation, Ambient and Free Vibration Testing

**Figure 6:**
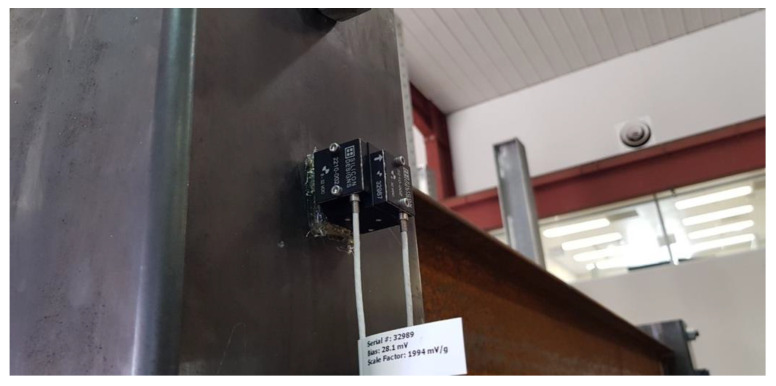
Accelerometers mounted at the top of module’s column.

The OMA procedure is adopted in this study to investigate the contribution of infill walls with different materials on the natural dynamic properties of the single steel module. In the OMA, it is not needed to record the input excitations, and just the output vibration response of the system is recorded. For recording the vibrations of the steel module, it is instrumented with six single-axis accelerometers. They are micro-electro-mechanical systems (MEMS technology) manufactured by Silicon Design Company (Kirkland, WA 98034 USA). High nominal sensitivity of 2 V/*g* makes them appropriate for applications where the level of accelerations is low as it is common in civil engineering structures. They have full scale range of 2 *g*, output voltage of ±16V, and output noise of 10 μ*g*/(root Hz). The accelerometers are installed at the top of module’s columns along the middle axis of ceiling beams through accompanied mounting kits as shown in Figure 6.

**Figure 7:**
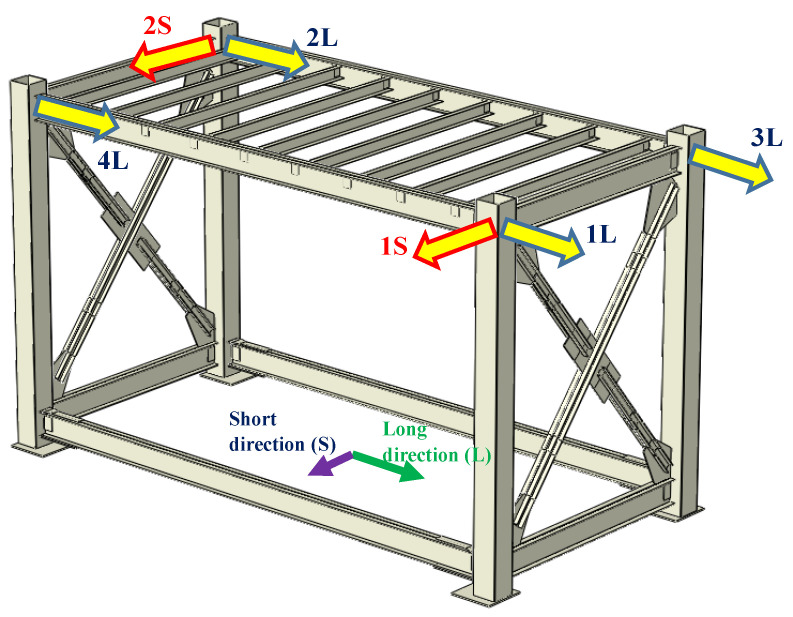
Position and orientation of accelerometers, along with the naming convention. Module plan. L: long direction; S: short braced direction.

Four accelerometers were oriented along the module’s long bay and named as 1L to 4L, and the remaining two were oriented along the short bay with the name of 1S and 2S. The orientation and naming convention of accelerometers are shown in Figure 7. The data acquisition system consists of a multi-channel National Instrument (NI) data logger with shielded four-core low noise cables. The NI data acquisition system was supported by LabVIEW software (v.2017, NI, Sydney, NSW, Australia) to synchronously record the accelerations at the rate of 1 K sample per second. 

**Figure 8:**
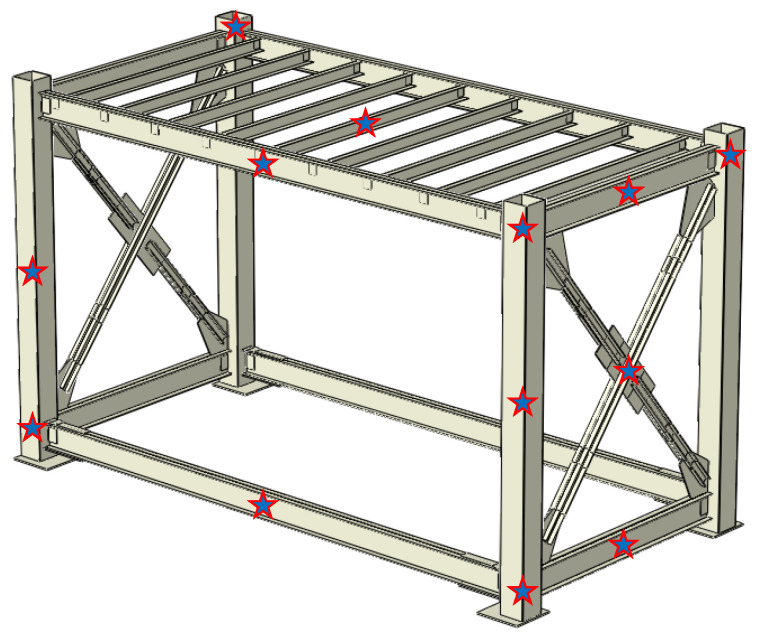
Locations of hammer impacts for free vibration testing of the module.

As input for the operational modal testing, the ambient accelerations of the system were recorded for the bare (frame only) module, and then for the different walled cases of II, IE, and EE, listed in Table 1. For exciting different natural modes and better capturing, in addition to the pure ambient vibrations, the module is impacted randomly with a rubber hammer at different locations and its free vibration response is recorded. These locations are bottom, middle and top points of the columns, middle points of the floor and ceiling beams, middle of the braces, and the middle point of the ceiling joists as representatively shown in Figure 8. These tests are repeated for all testing cases. The artificial impacts are of different intensities and orientations such that different modes of the bare module, and coupled module-wall system to be excited. The hammer impacts may produce acceleration orders of 0.01 *g*, 0.1 *g* and 1 *g*, such that the contribution of the wall system can be examined at different shaking levels. During ambient and free vibration tests, the acceleration response of the steel module is recorded for 5 s, with 5K sampled data. A low-pass filter with a cut-off frequency of 200 Hz was applied to the data to remove undesired noises.

## 4. System Identification

**Figure 9:**
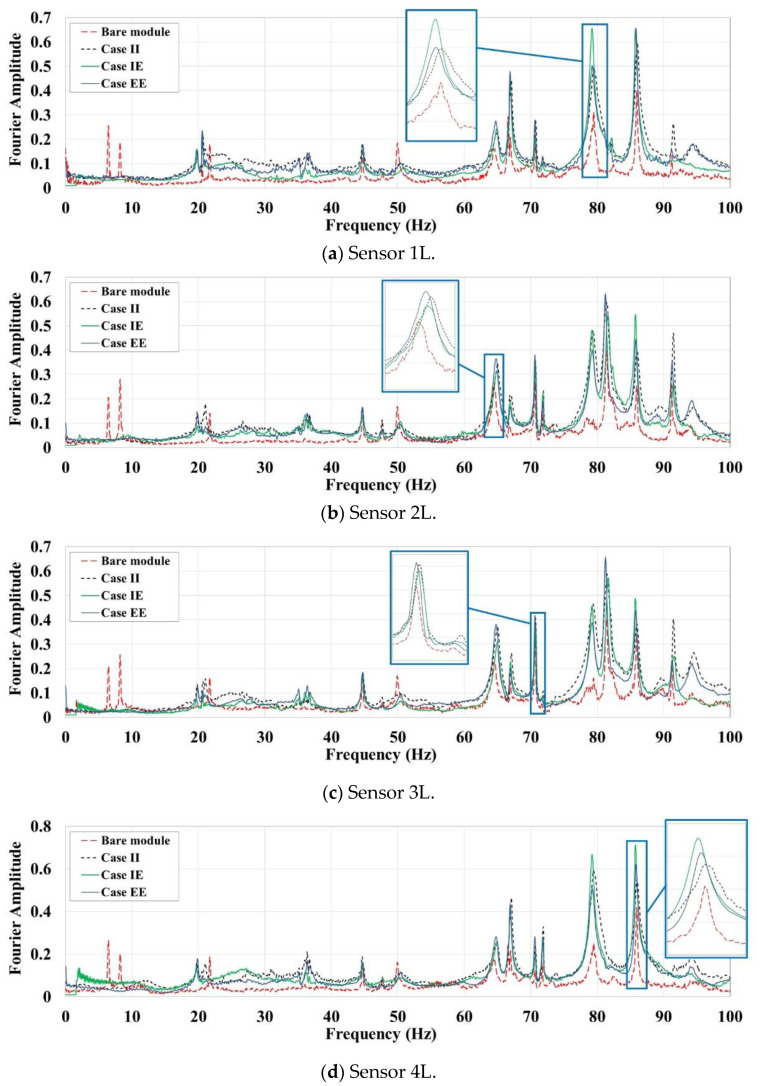
Averaged FFT plots for the L-oriented sensors.

**Figure 10:**
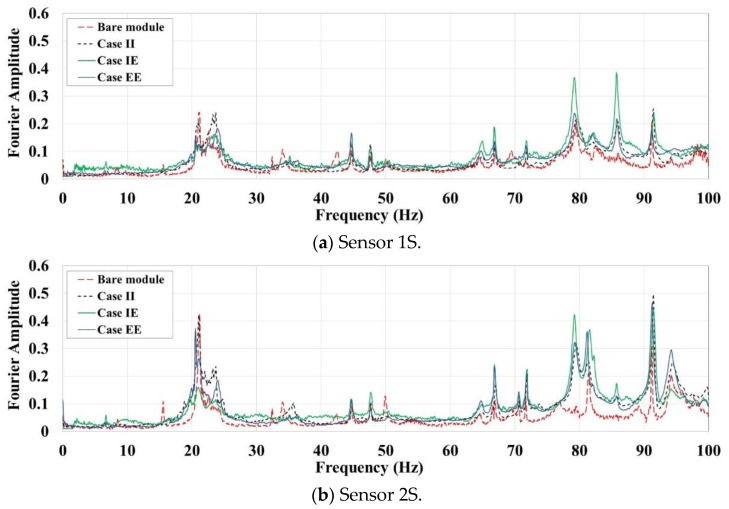
Averaged FFT plots for the S-oriented sensors. (**a**) Sensor 1S; (**b**) Sensor 2S.

One of the simplest methods for extracting the natural frequencies is peak-picking via direct Fast Fourier Transform (FFT) of the output responses. As the pure ambient and free vibrations responses include the natural characteristics of the examined system, the peaks of the FFT plots can be indicative of natural frequencies. As several tests of various intensities have been conducted on the steel module in different cases, their FFTs can be employed for system identification. To facilitate the comparison between different cases, distinct FFTs can be first normalised with respect to the maximum Fourier amplitude, and then averaged through different tests. The resulted averaged FFT plots are shown in Figure 9 and Figure 10 for the L- and S-oriented acceleration sensors, respectively.

Figure 9 shows the sensors oriented along the module’s long side, with the first peaks observed below 10 Hz for the bare module as fundamental translational modes; however, these peaks are removed for the coupled systems. The first peak of the coupled systems is observed at near 20 Hz for all cases. It shows a significant change of the steel module’s fundamental natural vibration mode, which is translational along the long direction when the composite LSF wall system is added. The bare module’s next peak is at 21.7 Hz, while the coupled system is observed at 20.6–21 Hz and 35.5–36.5 Hz with a little shift. A spike at 44.8 Hz for all bare and coupled cases shows that it can be related to a torsional mode with considerable effect from the shorter braced direction, which has the same detailing in all cases. Higher peak at 49.9 Hz is observed just for the bare module, while there are several high spikes above 60 Hz, which are observed for almost cases. It indicates that these modes have modal shapes dominated by movement along the shorter (braced) bay, which has similar properties across all test cases. It should be noted that the peaks of coupled system with different wall layout are located close to each other with slight shifts of maximum 0.4 Hz. This shows that when the module is covered with LSF wall systems, the infill panel material has negligible effects on the natural frequencies.

For the S-oriented sensors along the short direction in Figure 10, almost the same peaks are observed for all test cases. The little differences can be mainly related to the presence of the infill walls not to their material. As it is expected, adding partitions in one direction has negligible effects on the frequencies of another direction. Also, concurrent peaks in averaged FFT plots of L- and S-oriented sensors, essentially above 40 Hz, may be an indication of modes with torsional shapes that have components along with both module’s perpendicular main directions. The peaks related to translational modes of the system along the long direction are not observed in the FFT plots of the S-oriented sensors.

**Figure 11:**
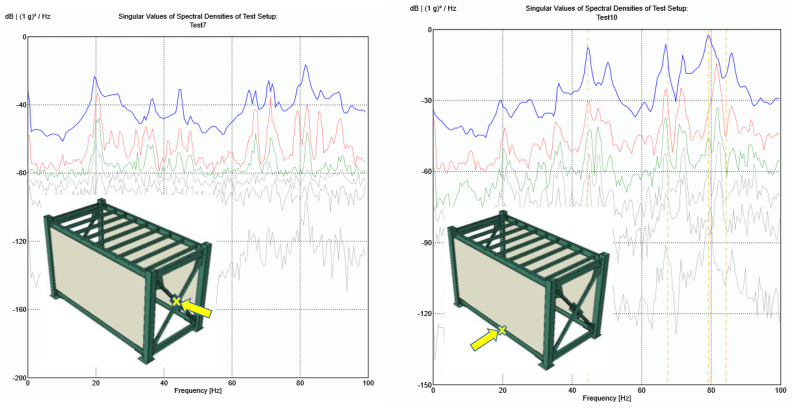
SVD diagrams of the recorded accelerations during two hammer impact tests on the EE test case.

In addition to the direct FFT, the output-only EFDD method can be also used to identify the natural frequency, mode shapes and modal damping ratios. In the EFDD method, the relationship between the unknown input *u*(*t*) and the measured responses *v*(*t*) can be expressed as [28]:

[*G_vv_*(*jω*)] = [*H*(*jω*)]^*^[*G_uu_*(*jω*)][*H*(*jω*)]^T^ (1)

where *G_uu_*(*jω*) is the *p* × *p* power spectral density (PSD) matrix of the input, *p* is the number of inputs, *G_vv_*(*jω*) is the *q* × *q* PSD matrix of the responses, *q* is the number of responses, *H*(*jω*) is the *q* × *p* frequency response function (FRF) matrix, and superscript T and ∗ denotes the transpose and complex conjugate, respectively [29,30,31,32]. This equation may be solved after mathematical manipulations that reduce the output PSD to a pole/residue form, and performing Singular Value Decomposition (SVD) of the spectral density matrices [12]. Application of the EFDD method on the recorded accelerations is conducted through ARTeMIS Modal Pro software [33]. The SVD diagrams for two hammer impact tests on the EE case (two exterior cement-board walls) are representatively shown in Figure 11. The natural frequencies and modal damping ratios are extracted and averaged for ambient and free vibration tests of all cases, and are listed in Table 2.

Similar results, as observed in the direct FFT plots, are obtained via the EFDD method concerning the natural frequencies. Considerable change is observed for the fundamental frequency, however, for the higher modes, less than 5% difference is observed through the test cases. Adding infill panels with different materials may cause small increase or decrease in the higher natural frequencies. This two-fold change is mainly because of the balance between additional stiffness as well as mass that the infill wall panels add to the steel module. By increasing the mode number, the modal damping ratios generally decrease. This value falls below 1% for modes with natural frequencies more than 40Hz in all the test cases. 

Based on the obtained results from experimental program, the module’s fundamental natural period, which is an important parameter in the quasi-static and dynamic analysis of MSFs, may dramatically change (even over 100%) when the non-structural and non-load bearing LSF composite infill walls are mounted. This level of change, which is not sensitive to the infill panel material, can totally alter the dynamic demands, and is not recognised by design standards and codes of practice [34]. Modal damping ratios may increase or decrease by adding the LSF composite walls, and no specific trend can be observed for the effects of infill panel material.

## 5. Finite Element Numerical Simulation

**Figure 12:**
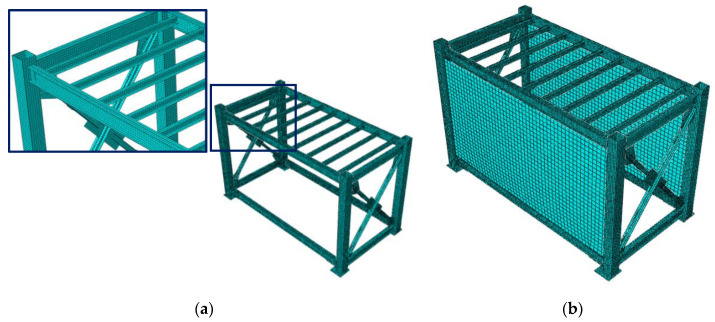
Numerical finite element model of (**a**) bare module, (**b**) coupled module-wall system.

Based on the experimental results, a numerical model for the module can be calibrated. The numerical model is constructed using the finite-element method at a micro level. For the steel module, its different parts are generated with their exact geometry using linear continuum solid elements as the drawing details in Figure 1. Because different parts are welded together, they are tied to each other at their interfaces in the numerical model. The numerical finite element model of the bare module is shown in Figure 12a. The properties of hot-rolled steel sections, i.e., SHS, UB and PFC, are considered with density of 7850 kg/m^3^, elastic modulus of 210 GPa and Poisson’s ratio of 0.3. The same properties are considered for the steel plates, which are made from Grade 350 structural steel. 

The parts made from cold-formed steel sections, i.e., the ceiling joists, tracks and studs, are modelled using shell elements, because their thickness is very low, less than 2 mm. Since the cold-formed steel sections are screwed, and connected firmly to the steel frame and each other, they are tied to the module in their interfaces. However, other boundary conditions may be considered in the calibration process. The properties of cold-formed steel material are assumed similar to the hot-rolled steel. All steel materials are considered as homogenous and isotropic.

For the purpose of model calibration, only the bare module as well as the module covered with interior walls, i.e., Case II, are numerically simulated. The gypsum sheathing plasterboards are modelled using linear thin shell elements, and tied to the tracks and studs. Two gypsum sheets are attached to both sides of the studs in each bay. The gypsum material properties are considered as homogenous and isotropic with density of 510 kg/m^3^, elastic shear modulus of 2.4 GPa, and Poisson’s ratio of 0.25. The coupled steel module-LSF composite wall system of the test Case II is shown in Figure 12b. 

The primary analysis step is frequency extraction, a linear procedure that computes the natural frequencies and mode shapes of the considered system. The module’s base plates are completely fixed during the frequency extraction analysis. The ABAQUS software package [35] was used for the purpose of numerical modelling.

**Figure 13:**
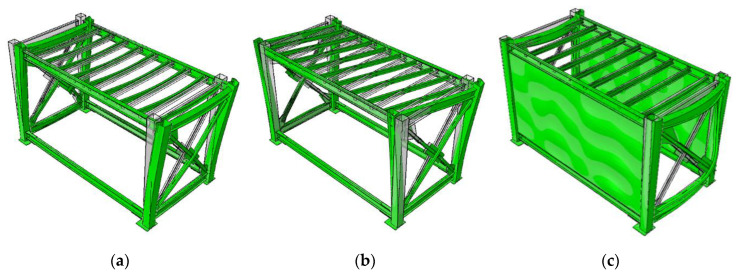
Fundamental natural vibration modes of (**a**) bare module, 1st at 6.6Hz; (**b**) bare module, 2nd at 8.0Hz; (**c**) coupled module-wall system, 1st at 18.5 Hz.

The first natural modes of the bare module, holding considerable effective mass, are computed as 6.6 Hz and 8.0 Hz, which show less than 3% deviation from the experimental results. The related mode shapes shown in Figure 13a,b. These natural modes have just movement component along the long side of the module, as it was predicted via experimental results. The fundamental natural mode of the coupled module-wall system is computed as 18.5Hz which differs from the experimental value about 6%. It has a translational mode shape along the long side of the module as shown in Figure 13c. More discrepancies are observed for higher modes, but the numerical model shows excellent agreement with the experimental results particularly for the fundamental natural mode. 

It is noted that agreement for the fundamental mode in the long direction is sufficient for this study, because of the system geometry, which resembles a single-storey building, and the importance of the fundamental natural mode for design purposes. Hence, the numerical model can be considered as calibrated. It indicates that in the linear range, the tying boundary conditions between the wall and the module is a reasonable assumption for the numerical simulation of the coupled system. It should be mentioned that this assumption in the nonlinear range, for example under monotonic or cyclic lateral loading schemes, may not be reliable anymore. It is also the case for insignificancy of the infill panel material.

## 6. Equivalent Strut Macro-Model

Adding full LSF composite walls with sheathing panels significantly increases the fundamental frequency of the module mainly because of additional in-plane stiffness provided by the infill sheets. Preliminary analyses revealed that the influence of the wall’s light-steel frame is negligible. Although this increase is in the linear range, considering full bonding between the wall and the module, it can significantly affect the demand of the lateral loading in linear quasi-static and pseudo-dynamic analysis procedures. 

Since the single module resembles a single-storey building, it can be assumed as a single-degree-of-freedom (SDOF) system [36]. Considering the module as an SDOF in the long direction, in both states of with and without infill walls, dominated by its fundamental natural mode, the ratio between the first mode frequencies, *ω*, would be:

(2) ωcωb=kcmckbmb=kcmbkbmc

where *k* is the stiffness, *m* is the mass, and subscripts *b* and *c* indicate the bare module and the coupled module-wall system, respectively. The total mass of the bare module is *m_b_* = 1235 kg, while for the coupled system of the cases II and EE, it is *m_c_* = 1475 and 1735 kg, respectively. It is noted that both cases II and EE are symmetric along the long direction. Considering the first mode vibration frequencies with the effective mass equal to half of the total mass, the ratio of the lateral stiffness between the two models is *k_c_*/*k_b_* ≈ 9.4 and 11.5 for the cases II and EE, respectively. It shows that adding full LSF infill wall with sheathing panels can rise the lateral stiffness of the bare steel module about 10 times. 

**Figure 14:**
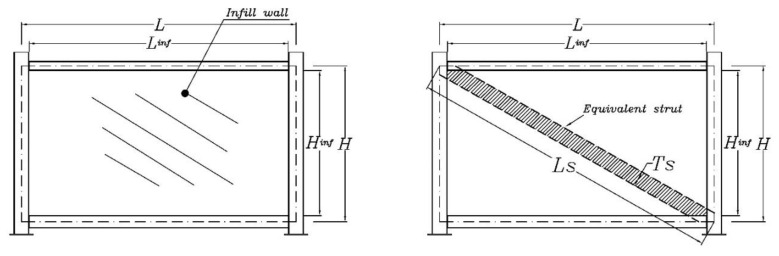
Schematic view of a general strut model for infilled modular frames.

It is not possible to propose a general strut model for LSF sheathed infill walls with a low number of experiments. But, in this study, a strut macro-model is proposed for the tested module and infill walls. As the experimental specimens were designed based on real conditions, fabricated full-scale and dynamically tested, it can provide insight for designers in practical applications. However, it should be mentioned that the proposed strut macro-model here is suitable for linear static and dynamic analyses, as it is based on the dynamic analysis in the linear range. Hence, it does not include nonlinear behaviour involving strength parameters or stiffness degradation. In the literature, the strut is usually assumed as a diagonal member with the same material and thickness as the infill, as shown in Figure 14. Many formulas have been suggested for its width, *Ts* [37]. Since most infill materials have low tensile strength, the strut typically woks in compression or possesses a nonlinear backbone force-displacement curve [38]. 

**Figure 15:**
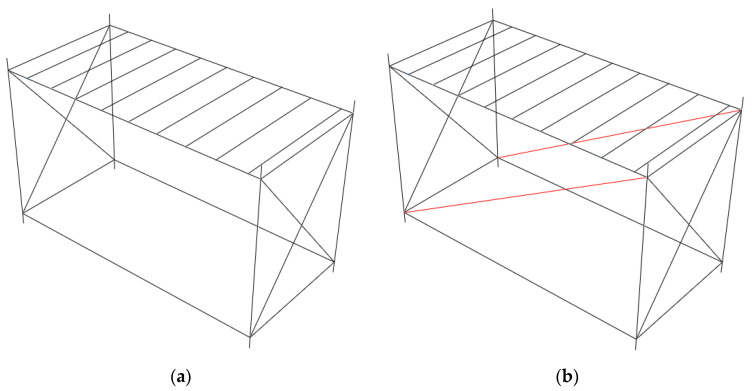
Numerical macro-model of (**a**) bare module, (**b**) module with added equivalent struts.

Because of the specific configuration of LSF composite walls, it is not straightforward to use the similar assumptions as equivalent struts of masonry infills. Therefore, here the infill wall is replaced with an equivalent steel strut; the main purpose is capturing the changes in the fundamental mode of the system through infills’ added initial stiffness and mass. The schematic macro-model of the module with and without strut is shown in Figure 15. The equivalent strut has linear behaviour recognized by its stiffness in tension. The lateral stiffness of the bare module, shown in Figure 15a, in the long direction, *k_b_*, considering pinned beam-column connections at the ceiling level, is mainly composed of flexural stiffness of the steel SHS columns. It can be calculated as:

(3) $$k_{b}=\frac{3EI}{l^{3}}\times n$$

where *E* is the steel Young’s modulus equal to 210 GPa, *I* is the moment of inertia of the columns equal to 39.2 × 10^6^ mm^4^, *l* is the column length equal to 2.6 m, and *n* is the number of columns which is four. By substituting these values, the lateral stiffness of the bare module is calculated as 5620 N/mm. 

**Figure 16:**
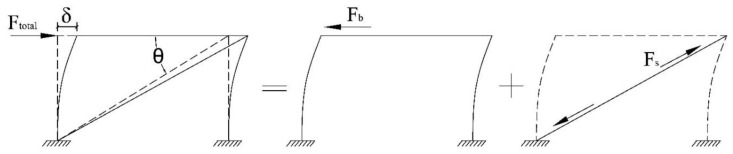
Separate contribution of module and strut in lateral load-bearing.

Then two struts are added to the module, one at each long bay to simulate the effects of infill walls, as shown in Figure 15b. Because they are hinged to the module frame, only their cross-sectional area is important parameter in their stiffness. From Figure 16, the following equation is obtained for the lateral loading of the coupled system:

(4) $$F_{total}=F_{b}+2F_{s}cos\theta$$

By dividing both sides of Equation (4) by lateral displacement *δ*, the lateral stiffness, *k_c_* is obtained as follows:

(5) $$k_{c}=k_{b}+2\frac{F_{s}}{\delta }cos\theta$$

The strut’s deflection can be computed as:

(6) $$\delta_{s}=\delta cos\theta$$

Hence, the lateral stiffness of the coupled system, *k_c_*, is composed of the lateral stiffness of the module, *k_b_*, and added axial stiffness of the struts, *k_s_*, through:

(7) $$k_{c}=k_{b}+2k_{s}cos^{2}\theta$$

where *θ* is the angle between the strut and longitudinal beams. Assuming *k_c_* = 10 × *k_b_*, the axial stiffness of each strut for the steel module is calculated as 32,600 N/mm. Considering the same steel material for the struts as for the module, the struts’ area, *A_s_*, is calculated:

(8) $$A_{s}=\frac{k_{s}l_{s}}{E}=\frac{\left(32600\right)\cdot \left(5200\right)}{210\times 10^{9}}\times 10^{6}=808\;mm^{2}$$

where *l_s_* is the strut length. This area can be considered as a steel circular rod with the diameter of 32 mm. Comparing with the lateral stiffness of the bare module, the normalised stiffness of each strut with respect to the modular steel unit is about 6. Although, it was computed based on the assumptions of this study, but this value can be considered as a practical suggestion for normalised axial stiffness of the strut macro-model equivalent to sheathed LSF composite walls typically used in modular steel units of MSFs.

## 7. Conclusions

In this study, a volumetric modular steel unit was experimentally tested full-scale using OMA technique under pure ambient vibrations, and randomly generated artificial hammer impacts. The module was tested in different situations. First, it was tested in bare (frame only) conditions, then its edges were filled with infill walls. The infill wall system used was the LSF composite wall system in which the infill linings with different materials (gypsum and cement-boards) were attached to the cold-formed LSF. Different configurations of gypsum and cement-board infill walls were tested. The coupled module-wall system was instrumented with sensitive accelerometers, and its vibration responses were synchronously recorded through a data acquisition system. The main dynamic characteristics of the module were extracted using output-only algorithms.

The results of system identification showed significant change (even over 100%) in the fundamental natural vibration frequency of the steel module, when the non-structural and non-load bearing composite LSF wall system is added. In addition, it was found that when the module is covered with LSF wall systems, the infill panel material has negligible effects on the natural frequencies causing a small increase or decrease. It was also found that by increasing the mode number, the modal damping ratios generally decrease. Modal damping ratios may increase or decrease by adding the LSF composite walls, and no specific trend can be observed for the effects of infill panel material. 

The contribution of this study is assessing the effects f different infill wall materials on the dynamic characteristics of modular steel units, and proposing simple models for macro-analysis of infilled module assemblies. There are other important parameters that affect the dynamic properties of modular buildings. Investigating the influence of these parameters, such as the combined effects of infill panels and connections (both internal and the relationships between the modules) can be the subject of future research. 

## Figures and Tables

**Table 1:** Different configurations of infill walls studied in this research.

Case Identifier	Description	Infill Wall Layout
Case II	Interior walls on both long sides	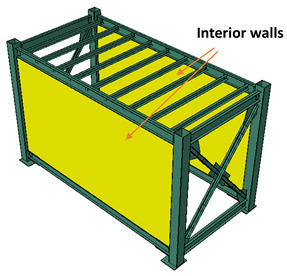
Case IE	Interior wall on one long side, and exterior wall on another long side	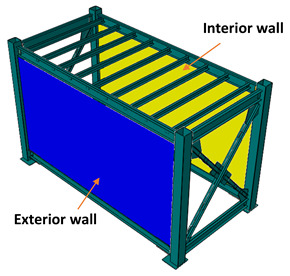
Case EE	Exterior walls on both long sides	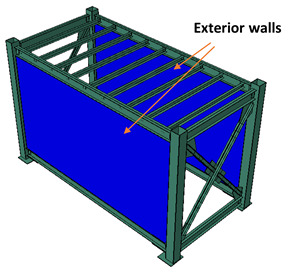

**Table 2:** Identified frequencies and modal damping ratios. The EFDD method.

Concurrent Mode	Bare Module		Test Case II		Test Case IE		Test Case EE	
	Freq. (Hz)	Damp. (%)	Freq. (Hz)	Damp. (%)	Freq. (Hz)	Damp. (%)	Freq. (Hz)	Damp. (%)
No. 1	6.4	4.5	19.7	2.1	19.7	1.6	19.8	1.8
No. 2	8.2	3.4	---	---	---	---	---	---
No. 3	21.7	1.5	21.7	1.6	20.9	1.7	20.8	1.5
No. 4	22.7	1.2	23.7	1.3	23.5	1.4	24.1	1.3
No. 5	34.1	0.9	36.1	1.1	36.5	1.3	36.6	1.1
No. 6	44.6	0.8	44.8	0.8	44.7	0.7	44.7	0.8
No. 7	49.9	0.8	---	---	---	---	---	---
No. 8	64.5	0.6	65.0	0.6	65.1	0.7	64.8	0.6
No. 9	66.6	0.6	66.9	0.4	66.8	0.4	66.6	0.7
No. 10	70.6	0.5	70.8	0.4	70.8	0.4	70.7	0.4
No. 11	79.5	0.6	79.5	0.8	79.3	0.9	79.0	0.6
No. 12	81.4	0.4	81.5	0.5	81.7	0.6	81.3	0.5
No. 13	86.0	0.3	86.1	0.4	85.9	0.6	85.9	0.5

## References

[1] Sharafi P., Rashidi M., Samali B., Ronagh H., Mortazavi M. (2018). Identification of Factors and Decision Analysis of the Level of Modularization in Building Construction. J. Arch. Eng..

[2] Sharafi P., Samali B., Ronagh H., Ghodrat M. (2017). Automated spatial design of multi-story modular buildings using a unified matrix method. Autom. Constr..

[3] Lacey A.W., Chen W., Hao H., Bi K. (2018). Structural response of modular buildings—An overview. J. Build. Eng..

[4] Alembagheri M., Sharafi P., Hajirezaei R., Tao Z. (2020). Anti-collapse resistance mechanisms in corner-supported modular steel buildings. J. Constr. Steel Res..

[5] Alembagheri M., Sharafi P., Hajirezaei R., Samali B. (2020). Collapse capacity of modular steel buildings subject to module loss scenarios: The role of inter-module connections. Eng. Struct..

[6] Quale J. (2017). Design in Modular Construction. J. Arch. Educ..

[7] Foraboschi P. (2016). Versatility of steel in correcting construction deficiencies and in seismic retrofitting of RC buildings. J. Build. Eng..

[8] Gorgolewski M.T., Grubb P.J., Lawson R.M. (2001). Modular Construction Using Light Steel Framing: Desing of Residential Buildings.

[9] Magliulo G., Petrone C., Capozzi V., Maddaloni G., Lopez P., Manfredi G. (2013). Seismic performance evaluation of plasterboard partitions via shake table tests. Bull. Earthq. Eng..

[10] Brincker R., Zhang L., Andersen P. Output-Only Modal Analysis by Frequency Domain Decomposition. Proceedings of the ISMA25 Noise and Vibration Engineering.

[11] Sharafi P., Mortazavi M., Samali B., Ronagh H. (2018). Interlocking system for enhancing the integrity of multi-storey modular buildings. Autom. Constr..

[12] Brincker R., Zhang L., Andersen P. Modal Identification from Ambient Responses Using Frequency Domain Decomposition. Proceedings of the 18th International Modal Analysis Conference (IMAC).

[13] Dawson R., Ward M.A. Dynamic Response of Framed Structures with Infill Walls. Proceedings of the Fifth World Conference on Earthquake Engineering (5th WCEE).

[14] Fang M.-J., Wang J.-F., Li G.-Q. (2013). Shaking table test of steel frame with ALC external wall panels. J. Constr. Steel Res..

[15] Yanev B., McNiven H.D. (1985). An Experimental Program for Studying the Dynamic Response of a Steel Frame with a Variety of Infill Partitions.

[16] Retamales R., Davies R., Mosqueda G., Filiatrault A. (2013). Experimental Seismic Fragility of Cold-Formed Steel Framed Gypsum Partition Walls. J. Struct. Eng..

[17] Gad E.F., Duffield C., Hutchinson G., Mansell D., Stark G. (1999). Lateral performance of cold-formed steel-framed domestic structures. Eng. Struct..

[18] Mortazavi M., Sharafi P., Kildashti K., Samali B. (2020). Prefabricated hybrid steel wall panels for mid-rise construction in seismic regions. J. Build. Eng..

[19] Usefi N., Sharafi P., Ronagh H. (2019). Numerical models for lateral behaviour analysis of cold-formed steel framed walls: State of the art, evaluation and challenges. Thin Walled Struct..

[20] Sharafi P., Mortazavi M., Usefi N., Kildashti K., Ronagh H., Samali B. (2018). Lateral force resisting systems in lightweight steel frames: Recent research advances. Thin Walled Struct..

[21] Mortazavi M., Sharafi P., Ronagh H., Samali B., Kildashti K. (2018). Lateral behaviour of hybrid cold-formed and hot-rolled steel wall systems: Experimental investigation. J. Constr. Steel Res..

[22] Restrepo J.I., Bersofsky A.M. (2011). Performance characteristics of light gage steel stud partition walls. Thin-Walled Struct..

[23] Macillo V., Bucciero B., Terracciano M.T., Pali T., Fiorino L., Landolfo R. (2017). 11.01: Shaking table tests on cold-formed steel building sheathed with gypsum panels. Ce Pap..

[24] Pnevmatikos N., Papagiannopoulos G., Papavasileiou G.S. (2019). Fragility curves for mixed concrete/steel frames subjected to seismic excitation. Soil Dyn. Earthq. Eng..

[25] Pnevmatikos N. (2012). New strategy for controlling structures collapse against earthquakes. Nat. Sci..

[26] Standards Association of Australia (SAA) (2012). S.G. Structural Design Actions—Part 2: Wind Actions.

[27] AS (1998). Steel Structures.

[28] Jacobsen N.-J., Andersen P., Brincker R. Using Enhanced Frequency Domain Decomposition as a Robust Technique to Harmonic Excitation in Operational Modal Analysis. Proceedings of the ISMA2006: International Conference on Noise & Vibration Engineering.

[29] Sevim B., Bayraktar A., Altunişik A.C., Atamturktur S., Birinci F. (2010). Assessment of nonlinear seismic performance of a restored historical arch bridge using ambient vibrations. Nonlinear Dyn..

[30] Kaveh A., Sharafi P. (2009). Nodal ordering for bandwidth reduction using ant system algorithm. Eng. Comput..

[31] Kaveh A., Sharafi P. (2008). Optimal priority functions for profile reduction using ant colony optimization. Finite Elem. Anal. Des..

[32] Kaveh A., Sharafi P. (2007). A simple ant algorithm for profile optimization of sparse matrices. Asian J. Civ. Eng..

[33] Structural Vibration Solutions (1999). Pro A.E. Release 4.1 Software.

[34] Standards Australia (2018). Structural Design Actions—Earthquake Actions in Australia.

[35] ABAQUS (2017). ABAQUS Theory Manual.

[36] Chopra A.K. (2001). Dynamics of Structures: Theory and Applications to Earthquake Engineering.

[37] Tanganelli M., Rotunno T., Viti S., Zampieri P. (2017). On the modelling of infilled RC frames through strut models. Cogent Eng..

[38] Mbewe P.B.K., Van Zijl G.P.A.G. (2019). Characterization of Equivalent Struts for Macromodeling of Infilled Masonry RC Frames Subjected to Lateral Load. J. Struct. Eng..

